# Post-LGM coastline evolution of the NW Sicilian Channel: Comparing high-resolution geophysical data with Glacial Isostatic Adjustment modeling

**DOI:** 10.1371/journal.pone.0228087

**Published:** 2020-02-03

**Authors:** Emanuele Lodolo, Gaia Galassi, Giorgio Spada, Massimo Zecchin, Dario Civile, Mathilde Bressoux

**Affiliations:** 1 Istituto Nazionale di Oceanografia e di Geofisica Sperimentale—OGS, Trieste, Italy; 2 Dipartimento di Scienze Pure e Applicate, Urbino University ''Carlo Bo'', Urbino, Italy; 3 Ecole Nationale Supérieure de Géologie, Université de Lorraine, Nancy, France; Universidade de Aveiro, PORTUGAL

## Abstract

Since about 20,000 years ago, the geography of the Earth has been profoundly modified by the gradual sea-level rise caused by the melting of continental ice sheets. Flat areas and regions characterized by very low gradients experienced, more than others, rapid flooding, with the progressive disappearance of vast coastal territories. Here we present a reconstruction of the late Quaternary coastline evolution of the north-western sector of the Sicilian Channel, constrained by high-resolution seismic profiles where the marker of the post-Last Glacial Maximum (LGM) marine transgression has been clearly identified and mapped. The locations of the post-LGM seismic horizon have been compared with predictions of a Glacial Isostatic Adjustment (GIA) model, which accounts for the migration of the shorelines in response to sea-level rise and for Earth’s rotational and deformational effects associated with deglaciation. We have verified that most of the points mapped through seismic data interpretation fall along the palaeo-coastline that the GIA model predicts for the 21 kyrs B.P. time frame. However, the model shows a misfit in the marine sector between Mazara del Vallo and Sciacca, where the available data indicate a Quaternary tectonic uplift. The analysis of the seismic profiles provides useful constraints to current GIA models. These add on existing histories of relative sea level in the Mediterranean Sea, allowing to gain new insight into the evolution of the palaeo-geography of the region of study and of the whole Sicilian Channel since the LGM, even in areas where direct geophysical observations are not available yet. In this respect, one of the most attractive implications of the ancient coastline evolution is linked with the underwater archaeology. The sea-level rise heavily impacted the distribution of human settlements, possibly forcing site abandonment and migrations, and this is particularly relevant in the Mediterranean basin, the cradle of the western civilization. The underwater traces left by these ancient populations represent the fundamental proofs to reconstruct the early history of our precursors.

## Introduction

It is well known that sea level has fluctuated throughout geological and anthropological time, periodically flooding or draining the coastal plains of the Earth. One of the time periods in which more detailed and constrained information on sea-level change are available, is that following the Last Glacial Maximum (LGM) about 20,000 years ago, when a relatively abrupt rise in sea level has occurred at a global scale, estimated to be 125 ±5 m [1–5]. A large amount of morphological studies, analysis of fossiliferous samples, and archaeological evidence have demonstrated that since the LGM the rates of sea-level rise have varied from less than 1 to over 40 mm/yr, as a result of melting of large continental ice sheets over north America, Europe, Siberia and Antarctica. The continuing adjustment of the solid Earth in response to the redistribution of ice and water masses is manifest through various geophysical phenomena. These have been studied to infer the extent and amount of the former ice masses, to reconstruct the sea level during a glacial cycle, and to constrain rheological properties of the Earth’s interior. Several models describing the Earth’s response to deglaciation have been developed [6–8] and used to predict shoreline evolution during a glacial cycle [9–11]. The most commonly used GIA models utilized to describe the response of the solid Earth, the gravitational field, and the oceans to the growth and decay of the global ice sheets, are reviewed in [12].

Tectonic processes that cause vertical movements (uplift or subsidence) of the coastal zones must be accurately analyzed and quantified because they result in an apparent sea-level fall or rise, and have therefore an important role in the interpretation of observed sea level. In most cases however, palaeogeographic reconstructions based on GIA modeling have mostly a qualitative character, due to (i) limited knowledge of the tectonic context of the area; (ii) partial and general low-resolution mapping of the sea-floor, and (iii) absence of age calibrations derived from wells and/or sediment coring. In these cases, the evolution of the ancient coastlines predicted by GIA models in response to sea-level rise offers only a general panorama of the modifications occurring for a given coastal area, which necessarily does not account for local phenomena and thus cannot be extended to larger areas. The available geological, geomorphological and archaeological evidence for the post-LGM period demonstrates this spatial variability, even the evidence is in most cases fragmentary. To realistically predict the evolution of the past shorelines, we need to understand the processes that lead to the complex patterns observed and quantify them. The availability of geological, structural and geophysical information for a specific area whose evolution is to be reproduced over time makes it possible to significantly refine the theoretical models, calibrate them appropriately, and make them as reliable as possible.

Here, for the first time, we propose a detailed palaeogeographic reconstruction of the western sector of the Sicilian Channel supported by a GIA model, which illustrates the sequence of the coastline evolution in various time frames, starting from the end of the LGM. In this area a new series of swath bathymetric data and high-resolution seismic profiles are available, on which the morphological and stratigraphic markers of the post-LGM transgression have been recognized. These markers offer the unique opportunity to test a GIA model against the new palaeogeographic scenarios proposed by the bathymetric data and to confirm its reliability and robustness, also dating the age of the beginning of sea-level rise in the absence of specific time calibrations.

One of the most attractive and intriguing implications of the persistent rise of the sea level that started about 20,000 years ago with the inception of the collapse of the last ice sheets are those related to the major changes within lifetimes and human memory of coastal dwellers. This global phenomenon has profoundly affected the history, social organization, and activities of ancient coastal populations, most probably triggering massive migrations.

## General geomorphological setting

**Fig 1:**
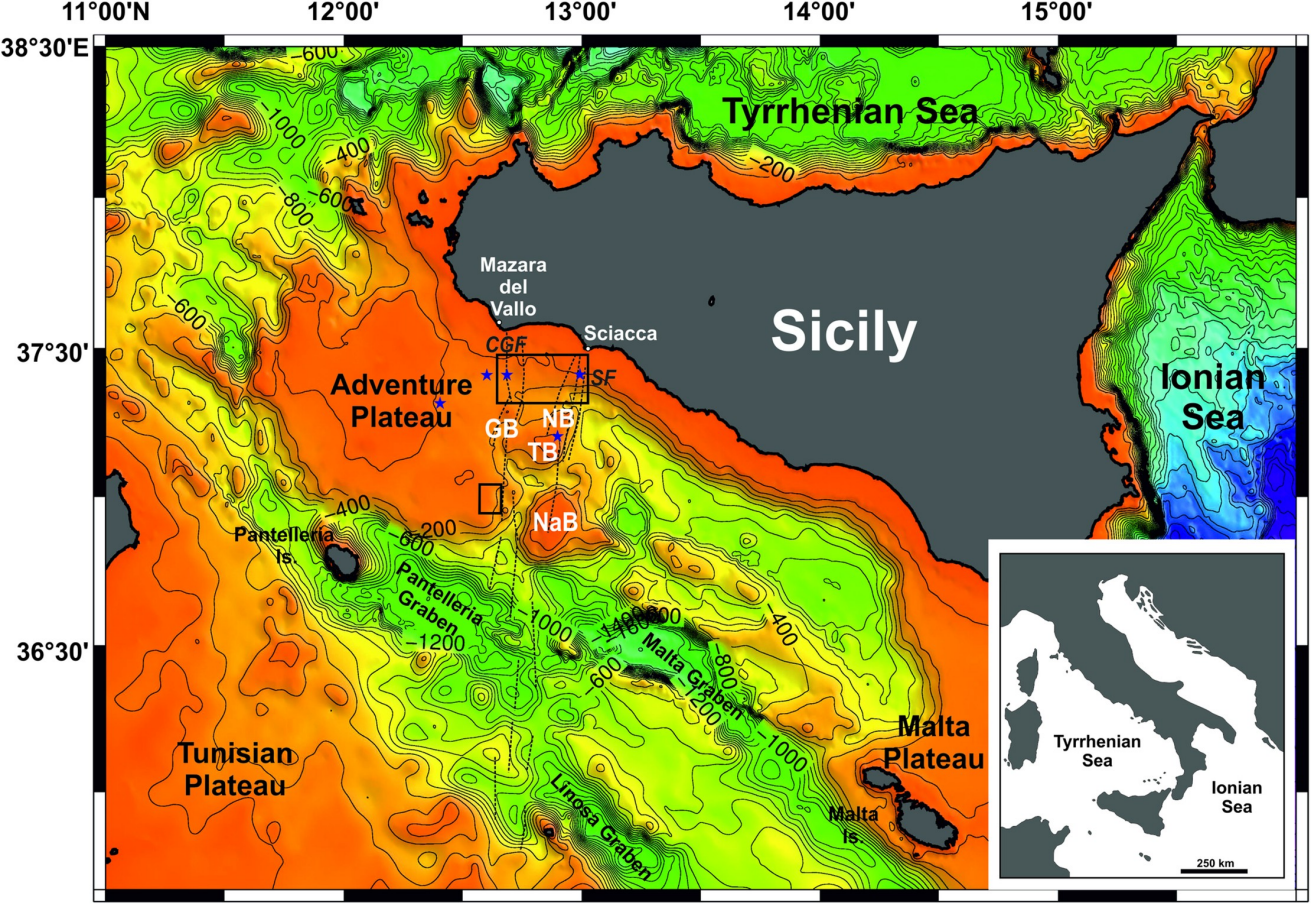
Physiographic map of the Sicilian Channel. Bathymetry taken from EMODnet Digital Terrain Model (1/16*1/16 arc minutes), downloaded from http://www.emodnet-bathymetry.eu/. Contours every 200 m. Abbreviations: GB, Graham Bank; TB, Terrible Bank; NB, Nerita Bank; NaB, Nameless Bank. Black rectangles indicate the areas where high-resolution bathymetric data are available (see Fig 2A and 2B). The dotted thin black segments indicate the main traces of the Capo Granitola (CGF) and Sciacca (SF) faults (modified from [27]). The small blue stars indicate the points where the Relative Sea Level (RSL) curves for the Adventure Plateau have been calculated (see Fig 6).

The Sicilian Channel is the submerged part of the northern Africa continental platform (Fig 1), lying mostly under shallow water (depths of less than 150 m), with the exception of three NW-trending, relatively deep troughs (the Pantelleria, Malta and Linosa graben). These tectonic depressions, which reach more than 1000 m in water depth, were generated since the early Pliocene by rift-related processes [13–16]. The shallowest parts of the Sicilian Channel are the Adventure Plateau in its NW sector, the Tunisian Plateau to the south, the Malta Plateau to the east and two areas of limited extent—the Nameless Bank and the area including the Graham (GB), Terrible (TB) and Nerita (NB) reliefs—located to the E of the Adventure Plateau. High-resolution marine geophysical surveys have shown that the Adventure Plateau, characterized by water depths ranging in general from 80 to 60 m, hosts several isolated banks of sedimentary and volcanic nature, some of them rising up to less than 10 m below sea level [17–19]. The Graham, Terrible and Nerita banks form a horseshoe-shaped relief open to the NW, where water depths range from 60 to 20 m, and located about 40 km off the coast of SW Sicily. Several volcanic edifices of different size and elevations are present in the Graham and Terrible banks [20], among them the ephemeral Ferdinandea Island.

Palaeogeographic reconstructions [18] have shown that during the LGM, the Adventure Plateau was connected to the former Sicily, forming a peninsula (the Adventure Peninsula) bulging toward south into the Sicilian Channel, and separated by the north African coastline by less than 50 km. The now submerged morphological highs identified in the Adventure Plateau, along with parts of the Graham, Terrible and Nerita banks, formed an archipelago of several islands until at least the early Holocene [21].

The NW foreland sector of the Sicilian Channel is traversed by a ~60 km long and 28–38 km wide, NE-trending lithospheric transfer zone [22–26] mainly developed during the Pliocene [26]. This lithospheric-scale structure is bounded by two strike-slip fault systems, i.e., the Capo Granitola Fault (CGF) to the west and the Sciacca Fault (SF) to the east (Fig 1), both dominated by positive flower structures. These two tectonic lineaments, developed as a right-lateral fault zones along preexisting (late Miocene) weakness zones, show significant evidence of Quaternary tectonic activity [27]. Studies have calculated that transpressional fold growth rates for both CGF and SF were high in latest Miocene-Pliocene and decreased during Quaternary [28].

## Geophysical data

**Fig 2:**
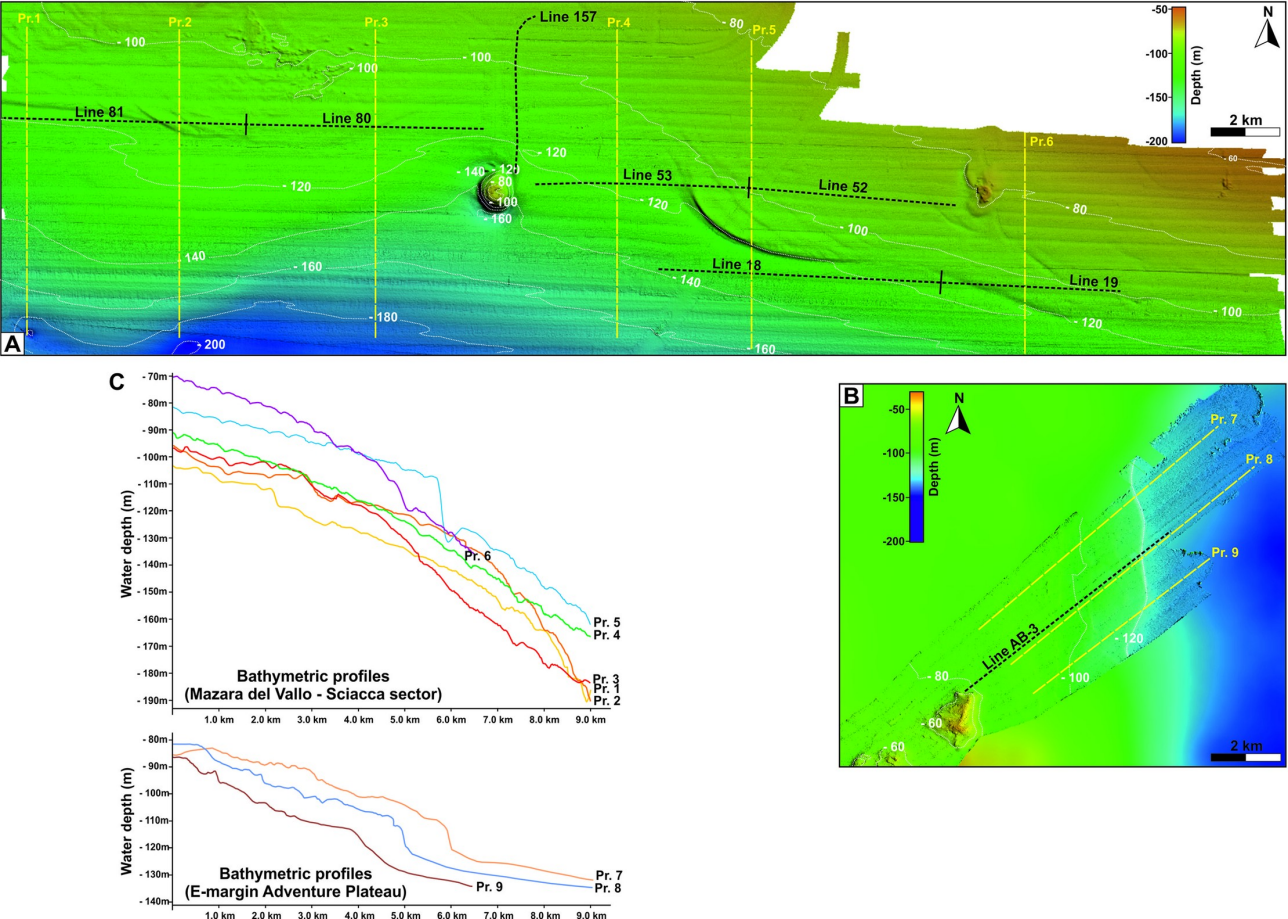
High-resolution bathymetry. Multibeam data of the Mazara del Vallo-Sciacca marine sector (A), and of the eastern margin of the Adventure Plateau (B) where the analysis has been carried out (see boxes in Fig 1). Positions of the corresponding Chirp profiles presented in Fig 4 are indicated. (C) Representative bathymetric profiles for the two analyzed sectors (corresponding locations in Fig 1A and 1B). The profiles were taken perpendicular to the maximum gradient associated to the post-LGM sea level rise. Data show that the bathymetric trends in the water depth ranging from 90 to 140 m are very different between the two areas taken into consideration. See text for details.

High-resolution swath bathymetry (Fig 2) and seismic profiles used in this study have been acquired during a series of geophysical surveys conducted on 2016 and 2018 in the NW sector of the Sicilian Channel onboard the R/V *OGS Explora*. Multibeam bathymetry has been realized with a keel-mounted Reson^®^ SeaBat 8111, operating at a frequency of 100 kHz and illuminating a swath on the sea-floor that is 150° across track and 1.5° along track. To ensure the best data coverage, every single swath was superimposed to the adjacent one for about one third. The resulting Digital Terrain Model, with a nominal resolution of 5 x 5 m, was visualized using the GlobalMapper^®^ software and the freely available GMT^®^ (Generic Mapping Tools). The high-resolution seismic profiles (Chirp profiles) have been acquired along the Multibeam swaths with a keel-mounted Benthos Teledyne^®^ Chirp III DSP-665, characterized by sweeps ranging from 2 to 7 kHz. Their vertical resolution is generally less than 1 m, with an acoustic penetration reaching 300 ms TWT (two-way travel time) in some areas where the sediment cover is particularly soft. Data were processed applying absorption compensation, migration and the Hilbert transform.

Our analysis of the high-resolution profiles is based on: (i) the identification and geometry of the prograding depositional bodies, (ii) the description of their seismic facies, and (iii) the analysis of the reflection terminations (erosional truncation, onlap, downlap). In this work, we are focusing on the water depth range between 100 and 140 m (corresponding to ~0.13 to 0.19 s TWT in the Chirp profiles), because this depth interval embraces the low-stand prograding wedge and therefore the LGM palaeo-shoreline. Available sedimentary cores have shown that the shallow banks of the Sicilian Channel are covered, for the most part, by a considerably reduced, unconsolidated bioclastic sand layer [29]. The basal mud of this sequence contains a neritic faunal assemblage, including benthic foraminifera. These sequences record Quaternary sea-level changes and thus, the development of a coarse calcareous sand layer over shallow water mud is related to the postglacial eustatic rise of sea level. Radiocarbon dating indicate that oceanographic conditions affecting the sea-floor changed significantly between the late Pleistocene and the early Holocene, and that non-deposition and/or erosion have prevailed since about 10,000 years B.P. [30]. This information has been used to correlate and calibrate the high-resolution seismic lines with the available cores, associating the acoustic reflectors with the corresponding lithostratigraphic levels and then convert TWT’s of the seismic lines to depths. Sound velocity measurements performed on the water column show that from 0 to -60 m, velocity decreases from 1520 to 1510 m/s, then from -60 to -90 m the sound velocity remains rather constant (1510 m/s), and from -90 to -180 m the velocity increments to 1514 m/s [18]. For extremely shallow, pore-saturated sediments, we applied in our time-to-depth conversion two extreme values of constant interval sound velocity (i.e., 1520 and 1600 m/s, respectively), and found that best results in the lithostratigraphic correlations were obtained applying a constant velocity of 1560 m/s. Taking into account the vertical resolution of the available seismic profiles, we estimate the errors in the depth conversions in about ±0.8 m.

**Fig 3:**
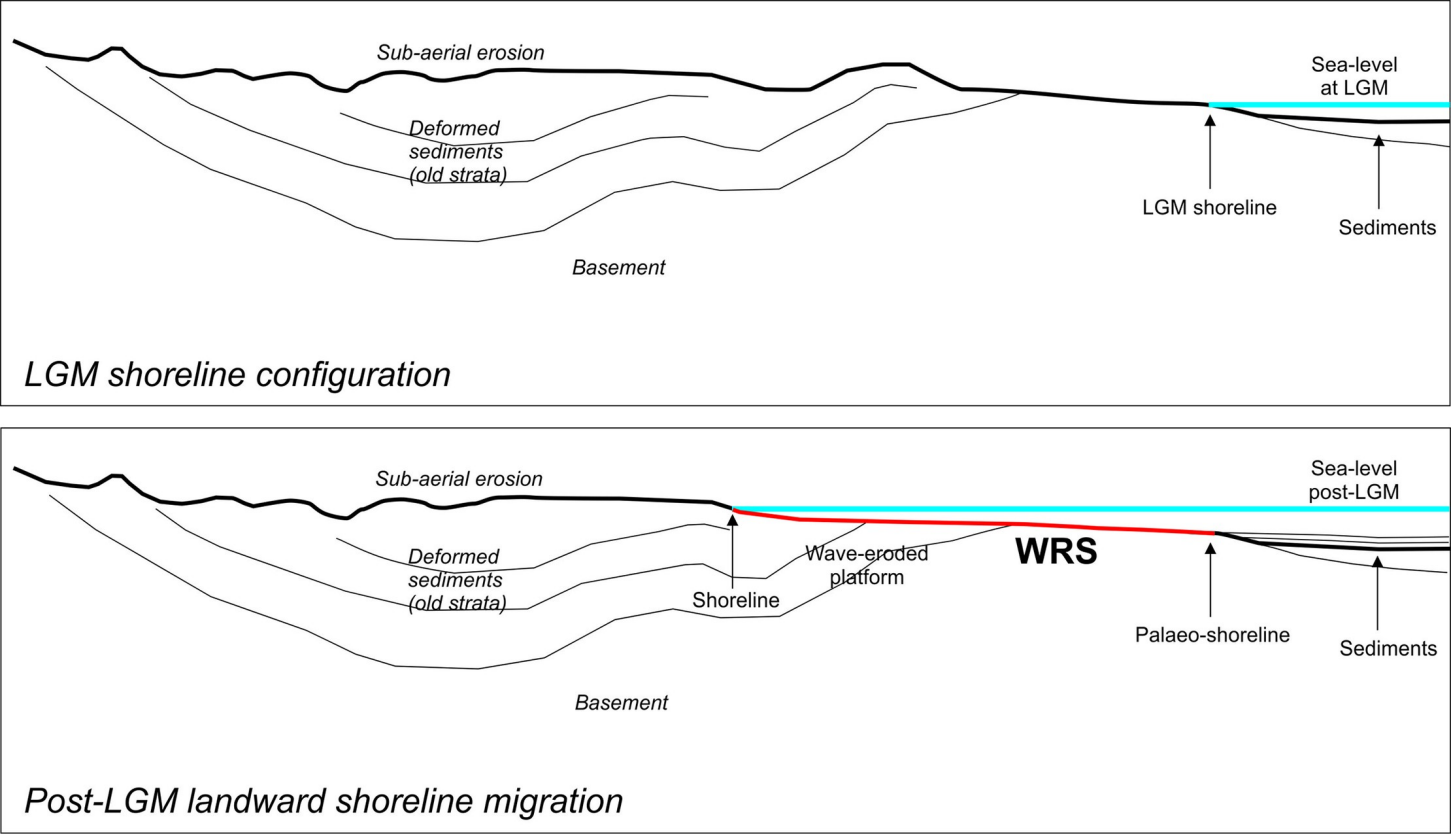
2-D simplified sketch of a low-gradient shelf. Shoreline configuration at the LGM (upper panel) and at a generic time-frame following the LGM where the sea level has rose (lower panel). The thick red segment indicates the "wave-ravinement erosional surface" (WRS).

The high-amplitude and generally continuous reflector which marks the exposed landscape at the time of maximum marine regression (i.e., the shelf-scale erosional unconformity related to the LGM) has been clearly identified on several Chirp profiles acquired in different parts of the NW Sicilian Channel. This unconformity represents the top of the low-stand prograding wedge (Fig 3). However, it should be noted that, with the exception of local deep valleys cut on the underlying deposits during the sea-level fall until the LGM, the subaerial unconformity may have been easily reworked by the "wave-ravinement surface" (WRS) [31], which represents the diachronous surface resulting from wave erosion during the post-LGM relative sea-level rise. WRSs are in general recognizable on seismic reflection profiles by: (i) their smooth, planar geometry over large horizontal distances; (ii) erosional truncation of underlying strata; and (iii) onlap of overlying strata [32–34]. The geometry of the WRSs produced during the post-LGM relative sea-level rise may vary from flat to very irregular due to several factors, the most important being the pre-transgression topography of the substrate, and the lateral variations in wave energy and bedrock resistance [32, 34–36]. In the case of low-gradient shelves, a barrier-lagoon system may develop during conditions of relatively slow sea-level rise which can be drowned and preserved during an episode of rapid or very rapid transgression, and later a new barrier develops landward during re-established conditions of slow relative sea-level rise ("in-place drowning" [36, 37]). For high-gradient shelves, a coastal cliff retreats due to eroding wave action at its toe during slow relative sea-level rise, and the WRS progressively migrates landwards. During rapid or very rapid relative sea-level rise, wave action has not enough time to completely dismantle the cliff, so the partially eroded cliff tends to be overstepped, and another coastal cliff may start to develop upslope during renewed conditions of slow relative sea-level rise ("cliff overstep" [35, 37]). Eventually, an infra-littoral prograding wedge (also known as submerged depositional terrace), starts to accumulate below storm wave base on the WRS [38].

**Fig 4:**
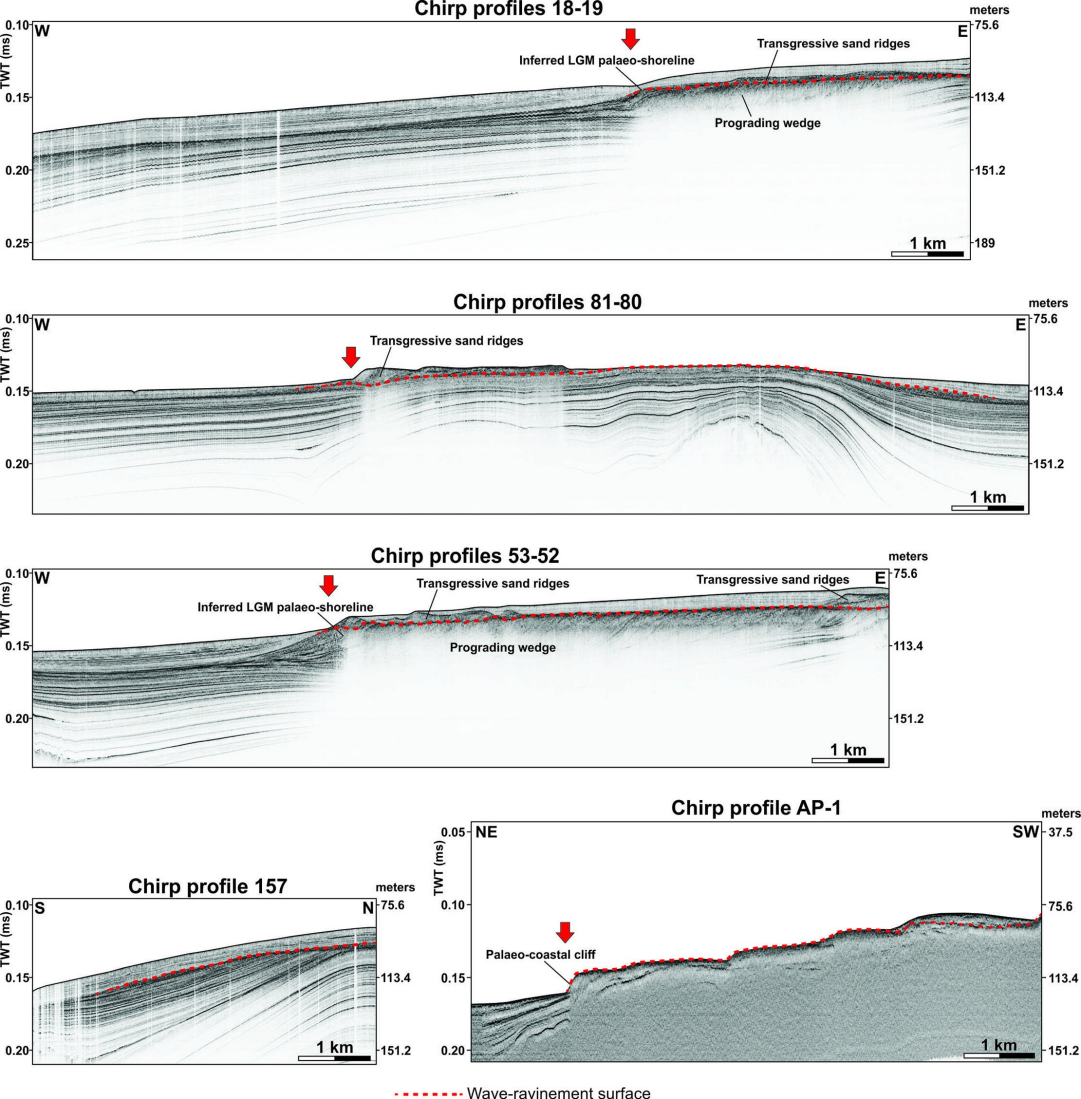
High-resolution seismic profiles. Representative Chirp profiles acquired off the SW coast of Sicily between Mazara del Vallo and Sciacca, and along the eastern margin of the Adventure Plateau (see Fig 2 for location). Red arrows indicate the locations of the post-LGM marine transgression. The depth vertical scale has been calculated applying a constant sea water sound velocity of 1512 m/s. See text for details.

The high density of the available Chirp profiles (spaced ~400 m apart) in the marine sector between the towns of Mazara del Vallo and Sciacca, allows us to identify the morphological and stratal features associated with the post-LGM transgression, and therefore to map in detail and with a good continuity the ancient coastline immediately before the marine drowning, that was located at that time ~15 km off the current one. Other sparse post-LGM points determined from Chirp profiles have been detected along the eastern margin of the Adventure Plateau, and in the northern part of the Nerita Bank. Here we show some representative profiles showing the general morphologies related to the post-LGM marine transgression along the sector between Mazara del Vallo and Sciacca, and one profile crossing the eastern margin of the Adventure Plateau (Fig 4). In general, the errors in depth derived from the picking of the horizons can be quantified numerically in ±0.2 m.

## GIA model and sea-level reconstructions

To model the time-evolution of the shorelines in the NW Sicilian Channel, we have solved numerically the "Sea Level Equation" (SLE), the integral equation describing the global sea-level variations induced by the melting of the late Pleistocene ice sheets. The SLE, originally formulated by Farrell and Clark [39], accounts for deformational, gravitational and rotational effects induced by time variations in the ice and in the meltwater loads, assuming a spherically symmetric Earth model with Maxwell viscoelastic rheology [40, 12]. To deal with "gravitationally self-consistent" time-variations of the Earth’s topography in response to GIA [7], we follow Milne and Mitrovica [41] and we employ a generalized formulation of the SLE, which accounts for the horizontal migration of shorelines, for the transition between grounded and floating ice during deglaciation, and for the effects of Earth rotation on sea-level change. Our numerical implementation of the generalized SLE has been successfully tested against other independently developed SLE solvers [42] and it has been recently used to study the dynamic evolution of the global coastlines since the LGM [43, 44].

**Fig 5:**
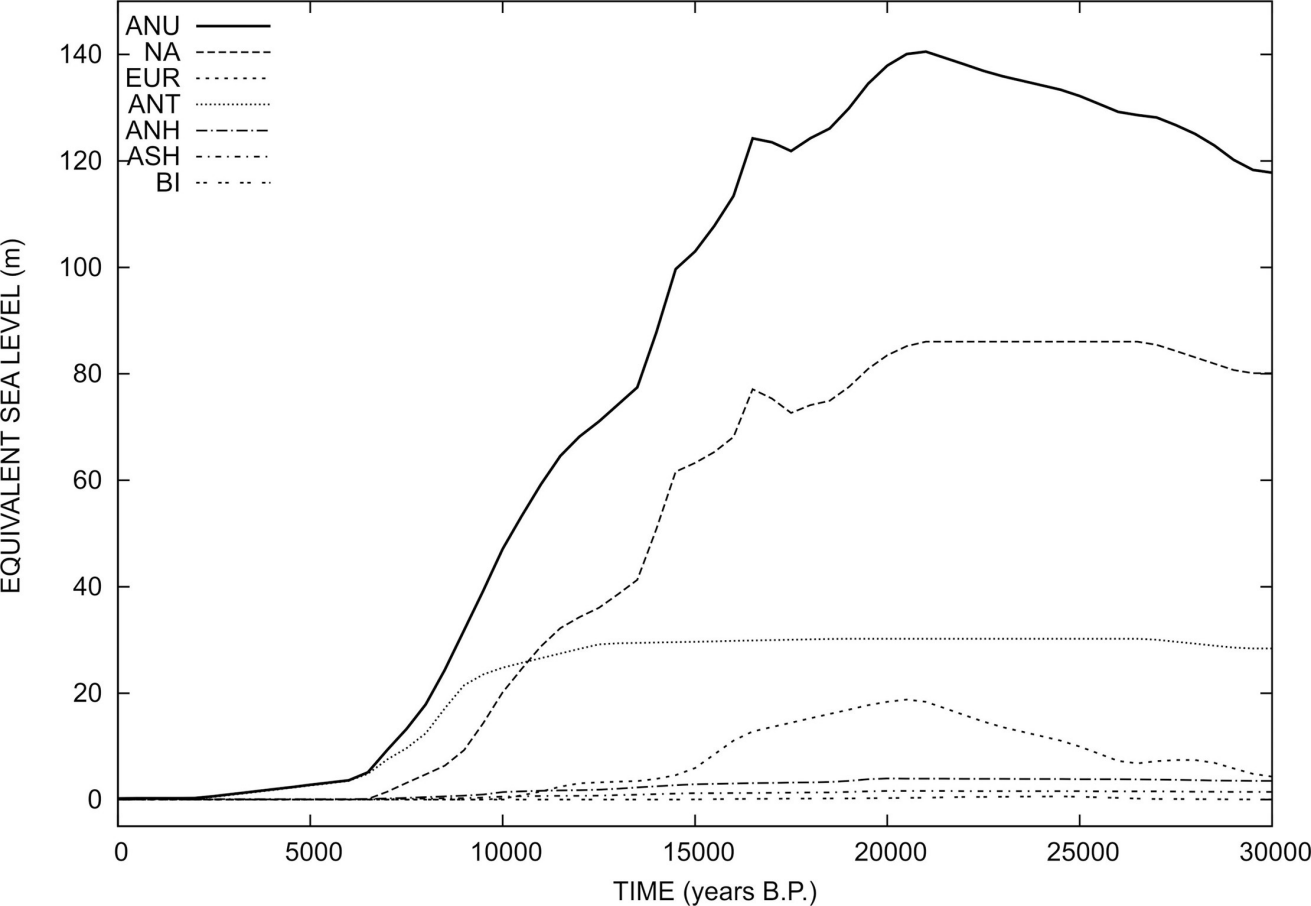
Equivalent Sea Level curves (ESL) for the GIA model during the last 30,000 years. The total ESL is marked by ANU (Australian National University); NA, North America (with Greenland and Iceland); EUR, Fennoscandia (including Barents-Kara); ANT, Antarctica; ANH, Alpine Glaciers (Northern Hemisphere); ASH, Alpine Glaciers (Southern Hemisphere); BI, British Isles. The three major components are clearly NA, ANT and EUR.

In this work, we employ the deglaciation chronology progressively developed by Kurt Lambeck and collaborators at the Australian National University–ANU [45–47]. The time history of the GIA model, which shall be referred to as ANU in the following, has been provided to one of us (GS) by Anthony Purcell in 2016. The ANU model assimilates the time evolution of the Northern and Southern Hemisphere ice sheets and glaciers during the last 30,000 years. This time frame is suitable for this study, which is focusing on the post-LGM period. The history of “Equivalent Sea Level” (ESL) for model ANU is shown in Fig 5 for all the ice bodies that compose the GIA model. By definition, ESL represents the globally uniform amount of sea-level change that would be observed for a rigid, non-gravitating Earth with fixed shorelines, i.e., ESL = (*ρ*i/*ρ*w)(Vi(t)/Ao) where *ρ*i and *ρ*w are the densities of ice and water, Vi(t) is the ice volume at time t and Ao is the present-day area of the surface of the oceans. The total ESL (solid line in Fig 5) reaches a maximum of ~140 m at 21,000 yrs B.P. (LGM), and vanishes approximately 1,500 yrs B.P. We note that during the last 7,000 yrs, the ESL variation is only due to the late contribution from the melting of the Antarctic ice sheet. The spatial distribution of the ice sheets and their time evolution in model ANU have been assimilated in our SLE solver SELEN^4^, an open-source program that simulates the GIA process in response to the melting of the late-Pleistocene ice sheets [48]. Using a pseudo-spectral approach complemented by a spatial discretization on an icosahedron-based spherical geodesic grid, SELEN^4^ solves a generalized SLE for a spherically symmetric Earth with linear viscoelastic rheology, taking the migration of the shorelines and the rotational feedback on sea level into account. The reader is referred to [48] for a very detailed description of SELEN^4^.

To reconstruct the global palaeo-bathymetry, we have followed the method proposed by Mitrovica and Milne [49], executing two nested iterations of the SLE, until convergence is attained. In the internal iteration, we solve the SLE for an a priori bathymetry by the pseudo-spectral method of Mitrovica and Peltier [50] utilizing the icosahedron-based geodesic grid of Tegmark [51]. In the external iteration, the bathymetry is updated using the global pattern of relative sea level (RSL) change obtained from the internal iteration. As a final constraint, we use the present-day topography according to the bedrock version of the one arc-minute resolution ETOPO1 global relief [52]. Based on previous work on the history of Holocene sea level along the Italian coast [4], we have adopted a Maxwell viscosity of 0.5 x 10^21^ Pa s in the upper mantle and in the mantle transition zone; the lower mantle viscosity and the lithospheric thickness are 10 x 10^21^ Pa s and 90 km, respectively. The elastic constants and the density profile are consistent with the PREM (Preliminary Reference Earth Model) seismological model of Dziewonski and Anderson [53]. The values of the model parameters are not changed throughout the paper, since they have been shown by Lambeck et al. [4] to provide a satisfactory fit to Holocene RSL curves in the Italian region. Furthermore, in the far field of previously glaciated areas the pattern of RSL change is more sensitive to the history of melting of the ice sheets than to the rheological parameters [53].

**Fig 6:**
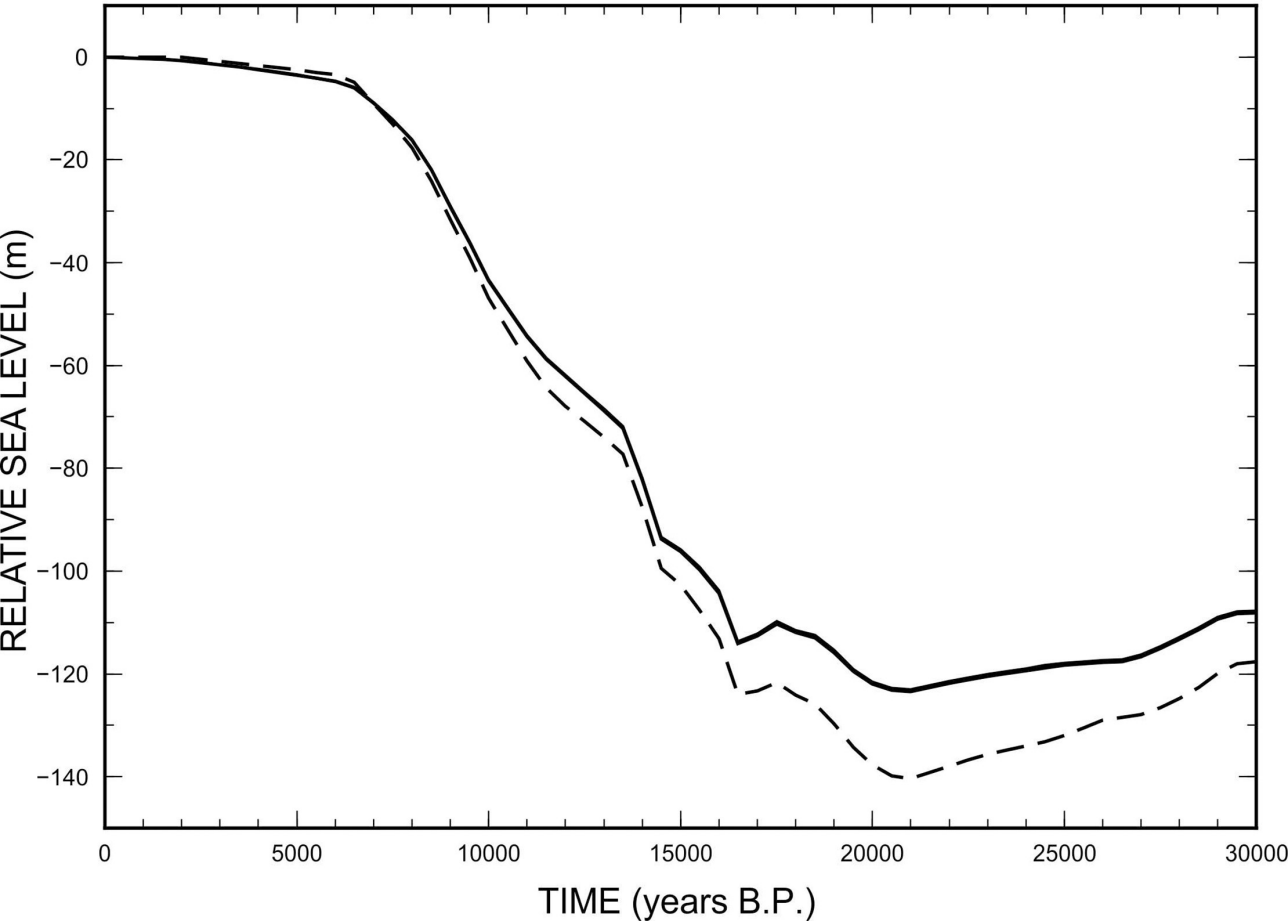
Relative Sea Level (RSL) for the Adventure Plateau. RSL curves calculated for the following points in latitude-longitude: 37.4°N 12.6°E; 37.4°N 13.0°E; 37.4°N 12.7E°; 37.3°N 12.4°E; 37.2°N 12.9°E (see locations in Fig 1). Note that the variations in sea-level in the various points are so small that the curves are practically overlapping and indistinguishable each other. The dashed curve is the eustatic curve for model ANU (Australian National University). The five curves differ by ~15 m at the LGM (21 kyrs).

**Fig 7:**
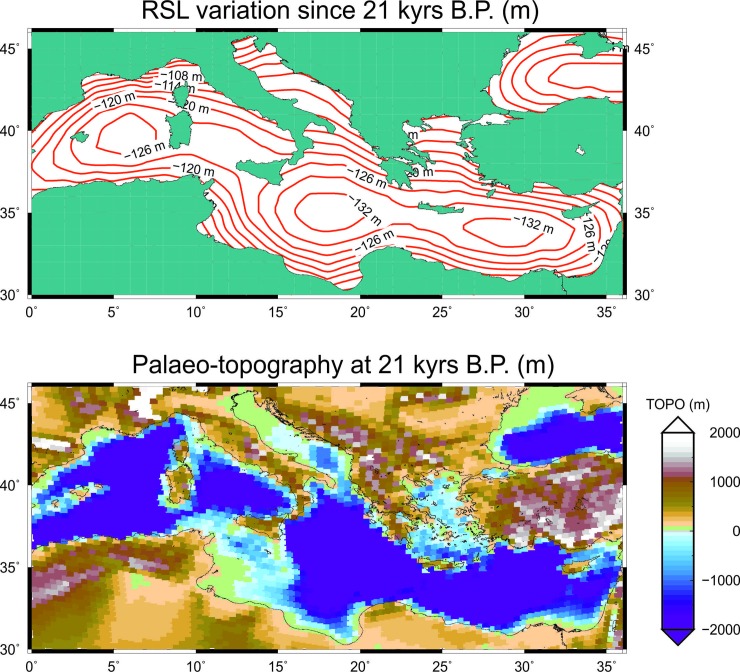
Relative sea level variation since 21 kyrs B.P. for the entire Mediterranean Sea obtained with SELEN4. The upper panel indicates the sea-level contours (in meters) at 21 kyrs B.P., the lower panel indicates the palaeo-topography at 21 kyrs B.P. (in meters).

The RSL curves generated by the model are shown in Fig 6 for five locations in the sector between Mazara del Vallo and Sciacca (the coordinates are given in the caption). The individual curves (solid lines) are practically indistinguishable from one another, as the variations in the sea-level trendare extremely modest for nearby points. We note sudden increases in RSL in the period between 14,000 and 13,000 yrs B.P. and between 16,300 and 15,500 yrs B.P., while the curves show an abrupt but short-lived RSL fall between 17,200 and 16,000 yrs B.P. These changes reflect those that characterize the eustatic curve, shown by the dashed line as a reference. However, it is also worth to note that the sea-level variation shown by the RSL curve at the five sites differs significantly from the eustatic curve, which confirms the importance of glacio-hydrostatic effects across the Mediterranean Sea despite the distance from the previously glaciated regions [1]. The modeled RSL variation since LGM across the Mediterranean basin and the palaeo-topography at the LGM are shown in the top and bottom frames of Fig 7, respectively. Despite the low-resolution, the topography map clearly shows the main areas exposed at the LGM, and points to a complex landscape in the Sicilian Channel. Throughout the paper, we shall only employ the ANU model, since it has been extensively calibrated by Kurt Lambeck and collaborators, using RSL data from the Mediterranean region (see [10] and references therein). A rigorous assessment of the GIA uncertainty associated with a different parameterization of the model is out of the purposes of this work. However, we know that once the history of deglaciation is given, uncertainty associated to the values of viscosity in the mantle can be considered small, as soon as we limit our analysis to sites that are sufficiently away from the previously glaciated areas [54].

**Fig 8:**
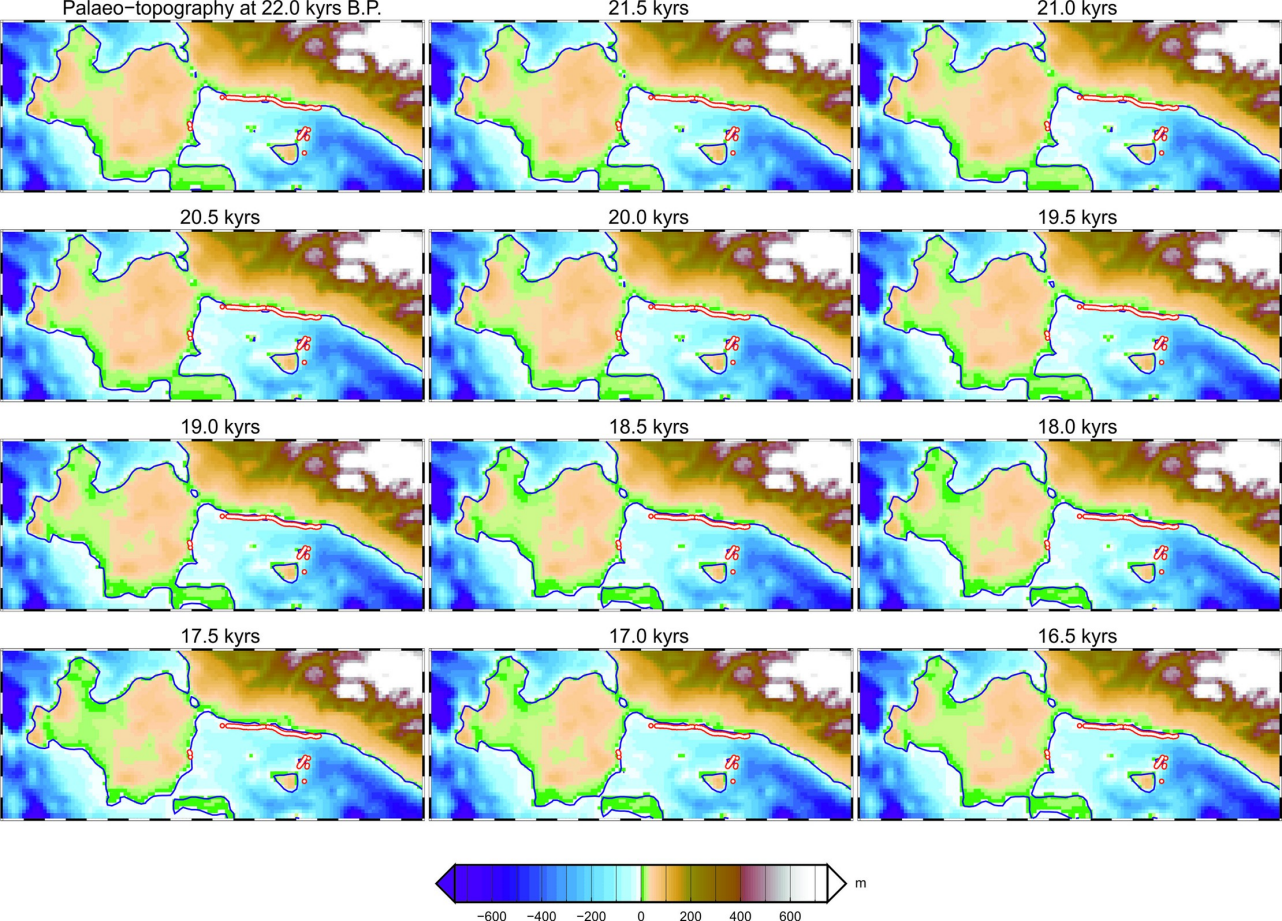
Evolution of the NW Sicilian Channel coastlines at different epochs. Maps obtained from the GIA model for the NW sector of the Sicilian Channel, showing coastline configuration every 500 years. The small red dots indicate the geographical positions of the beginning of the post-LGM marine transgression, as individuated from the analysis of the high-resolution seismic data.

The stratigraphic boundary between the WRSs and the onlap terminations of the overlying seismic reflectors provides the geometric constraints (i.e., the geographic location) for the proxy palaeo-shorelines as identified from all the available Chirp profiles. In Fig 8, these locations have been mapped along with the palaeo-coastline configurations obtained by GIA modeling for the NW sector of the Sicily Channel. The evolution of the coastlines is presented here in the form of twelve palaeogeographic maps covering a time span ranging from 22 to 16.5 kyrs B.P., at 0.5 kyrs intervals to focus the analysis on the time frame immediately following the LGM and evaluate, at least qualitatively, the match with the location of the LGM marker derived from the interpretation of the Chirp profiles.

## Results

We analyzed high-resolution seismic profiles along the NW margin of the Adventure Plateau, off the Nerita and Graham banks, and in the coastal sector comprised between the cities of Mazara del Vallo and Sciacca, where the seismic profiles are densely distributed and regularly spaced, and where we have a complete high-resolution bathymetric map available (see Fig 2). The seismic data have been used to identify the morphological elements associated with the landward coastline migration due to the effect of the post-LGM sea-level rise. This information is decisive for binding the temporal sequence of the coastline evolution produced by the models, and to calibrate the transgressive events in the absence of sampling and direct measurements. In the absence of tectonic vertical motions, the best fitting between the position of the coastlines produced by the GIA model in a given time-frame, and the coastline position deriving from the analysis of the seismic lines and the bathymetry, represents the constraint that defines the time at which sea level started to rise just after the LGM.

Chirp profiles show that the seismic units above and below the post-LGM horizon have seismic characters that in general change both laterally and vertically. This is clearly understandable considering that the region of study is extremely vast, with morphological, depositional, and structural characteristics varying significantly from place to place. Regarding the LGM palaeo-shoreline, it is inferred to have been located at the LGM clinoform rollover point, at the seaward termination of the subaerial unconformity [33]. The clinoform foresets are inferred to consist of shoreface deposits in their upper part, passing downdip into shelf deposits. However, based on the above, the original shoreline was likely reworked by the distal, earliest part of the WRS, and therefore its features are not always recognizable.

A prograding wedge is well-imaged in the Chirp profiles 18–19 and 53–52 (see Fig 4); it has a maximum thickness of ~0.03 s (~23.4 m) and is composed of top-truncated offlapping clinoforms consisting of foreset and bottomset that rapidly pinches out seaward. The erosional surface that truncates the top of the prograding wedge dips seaward, is locally irregular and ends roughly at the rollover point of the last (distal) clinoform, at a depth of ~0.14 s (~109.2 m). Such a surface is overlain by irregular ridges in places showing internal inclined strata, which are usually buried (excepting in the profiles 81–80 and AP-1) by gently seaward-dipping strata. Although the prograding wedge is not clearly visible in the Chirp profiles 81–80, 157 and AP-1, the erosional surface is still recognizable in some cases down to ~0.15–0.16 s (117.5–124.8 m). In the AP-1 profile, the irregular surface shows a stepped geometry and its distal part is steeply inclined seaward. The present evidence suggests that the erosional surface truncating the top of the prograding wedge is a WRS related to the post-LGM sea-level rise. The WRS is inferred to have reworked a previously formed subaerial unconformity [33, 34]. The LGM palaeo-shoreline has been placed close to the clinoform rollover point [33], although it was reworked by wave processes during transgression. The prograding wedge, therefore, can be interpreted as a shallow-marine sedimentary body that migrated during relative sea-level fall and lowstand. The ridges that overlie the WRS may be interpreted as sand ridges that migrated during the transgressive phase due to the presence of shelf currents. The steeply-inclined distal part of the WRS in the AP-1 profile probably represents a relict palaeo-coastal cliff developed during the LGM and then partially reworked at the onset of the transgression.

**Fig 9:**
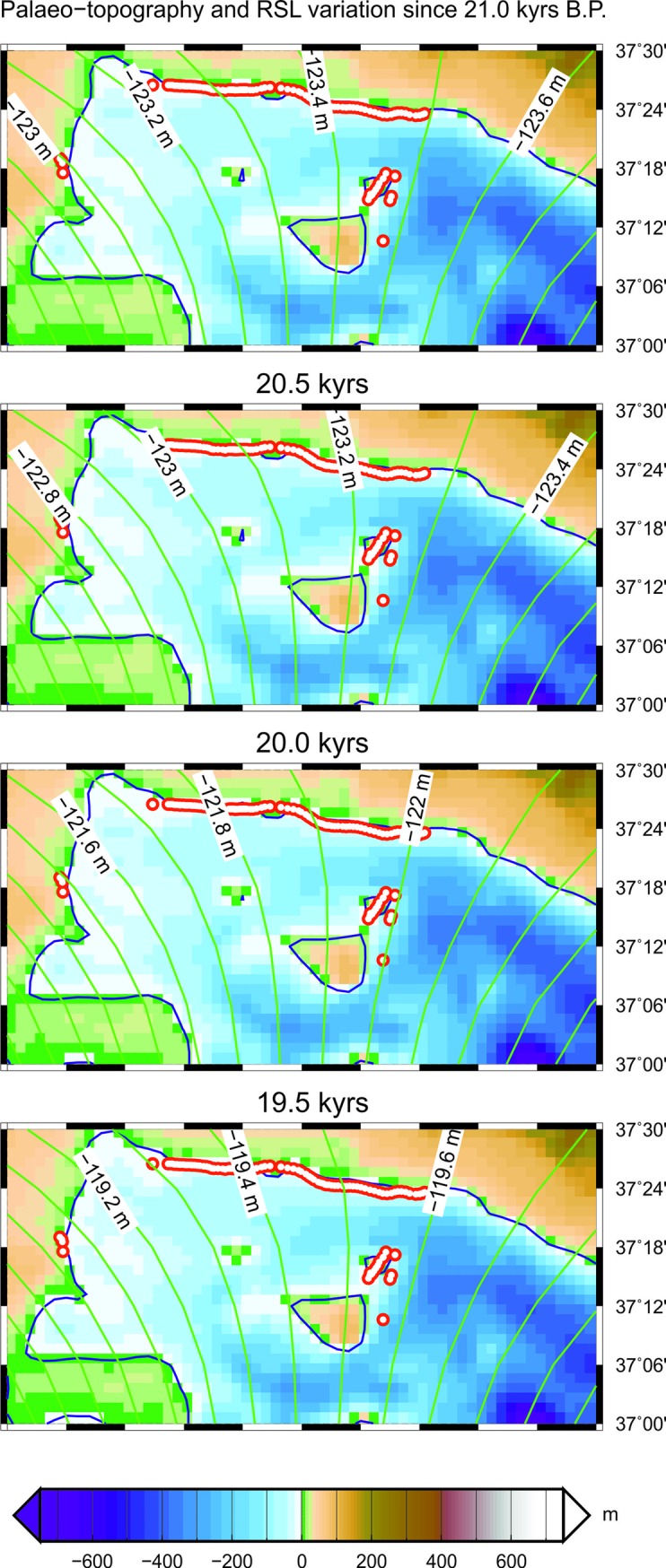
Palaeo-coastlines in the sector comprised between Mazara del Vallo and Sciacca from 21 to 19.5 kyrs B.P. In this sector, where the density of the available data is maximum, we have identified in detail the beginning of the post-LGM marine transgression (small red dots). The RSL curves for this sector have been also mapped (green lines).

The sector between Mazara del Vallo and Sciacca (Fig 9) is the one where it is easier and more immediate to visually appreciate the fit between the coastal profile produced by the GIA model and the one derived from the interpretation of the high-resolution seismic lines because of the significant density of available profiles. In Fig 9, along with the palaeo-topography, we show by contour lines the RSL variation (i.e., the depth of the sea relative to present) that is predicted according to the GIA model. The analysis of the bathymetric and seismic data indicates that the beginning of the post-LGM marine transgression is limited in a water depth range comprised between 105 and 110 m. The relatively small deviations in the values found along the various high-resolution seismic lines and the bathymetric profiles are attributable to several factors, as the picking errors (in terms of TWT) of the marine transgression surface, not always perfectly identifiable on the profiles, to the vertical resolution of the seismic lines, and to the presence of pre-existing morphological heterogeneities of the investigated area, which extends for more than 40 km. The data in Fig 2 show that the depth corresponding to the beginning of the post-LGM sea-level rise in the Mazara del Vallo-Sciacca sector is shallower of about 12 m with respect to the depth (RSL variation) expected by GIA modeling assuming tectonic stability for the area (i.e., in the hypothesis of no vertical motion of the ground in addition to that driven by hydro-isostasy). Assuming that our modeling in Fig 9 is providing a reliable scenario for the RSL variations associated with GIA, this implies that the area has undergone an uplift at a rate which may be quantified at ~0.6 mm/yr in the last 20,000 years. This value represents an average of the vertical tectonic rate of the studied area, derived from the analysis of the high-resolution seismic profiles since the LGM and by the adoption of a GIA model whose predictions are shown in Fig 9. As previously mentioned, this sector is crossed by the Capo Granitola and Sciacca faults, which are part of a lithospheric-scale, strike-slip system along a main N-S trending direction and crossing most of the Sicilian Channel. Available multi-channel seismic reflection profiles have shown that transpressional motion along both tectonic lineaments have developed remarkable flower structures [27], and calculated fold growth rates show decreasing values during the last ~1.8 Ma from 0.07 mm/yr (along Capo the Granitola folded structure) to 0.22 mm/yr (along the Sciacca folded structure), respectively [28]. Geological surveys and geodetic data show the occurrence of active deformation also in the onshore area of SW Sicily along thrust ramps [55]. On the contrary, the position of the beginning of the post-LGM marine flooding along the eastern margin of the Adventure Plateau as resulted from the Chirp data interpretation is compatible with the water depth at the LGM resulting from the GIA model, suggesting no significant vertical tectonic movements in this sector of the Sicilian Channel. The morphology of the sea-floor and the bathymetric profiles (see Fig 2) further testify that there are differences between the Mazara del Vallo-Sciacca sector and the eastern edge of the Adventure Plateau. In the first case, the post-LGM sea-level rise does not always have a well-defined morphological expression, while along the margin of the eastern Adventure Plateau there is a well-defined morphological step that clearly shows the beginning of the marine transgression. In addition, the morphological expression of the post-LGM marine transgression in the sector between Mazara del Vallo and Sciacca varies significantly from E to W: a very pronounced curved terracing is seen in the eastern part, and behind this morphological ridge, other but less pronounced undulations are detected from swath bathymetry. On the contrary, in the western part of the same marine sector, the morphological expression of the marine transgression is less evident: here, some elongated sedimentary lenses may be present, possibly associated to the activity of bottom currents. Differences in bedrock resistance to the erosion, local physiography and hydrodynamics may eventually also account for the observed lateral variability of the morphological elements under the same conditions of relative sea-level change [36, 56].

**Fig 10:**
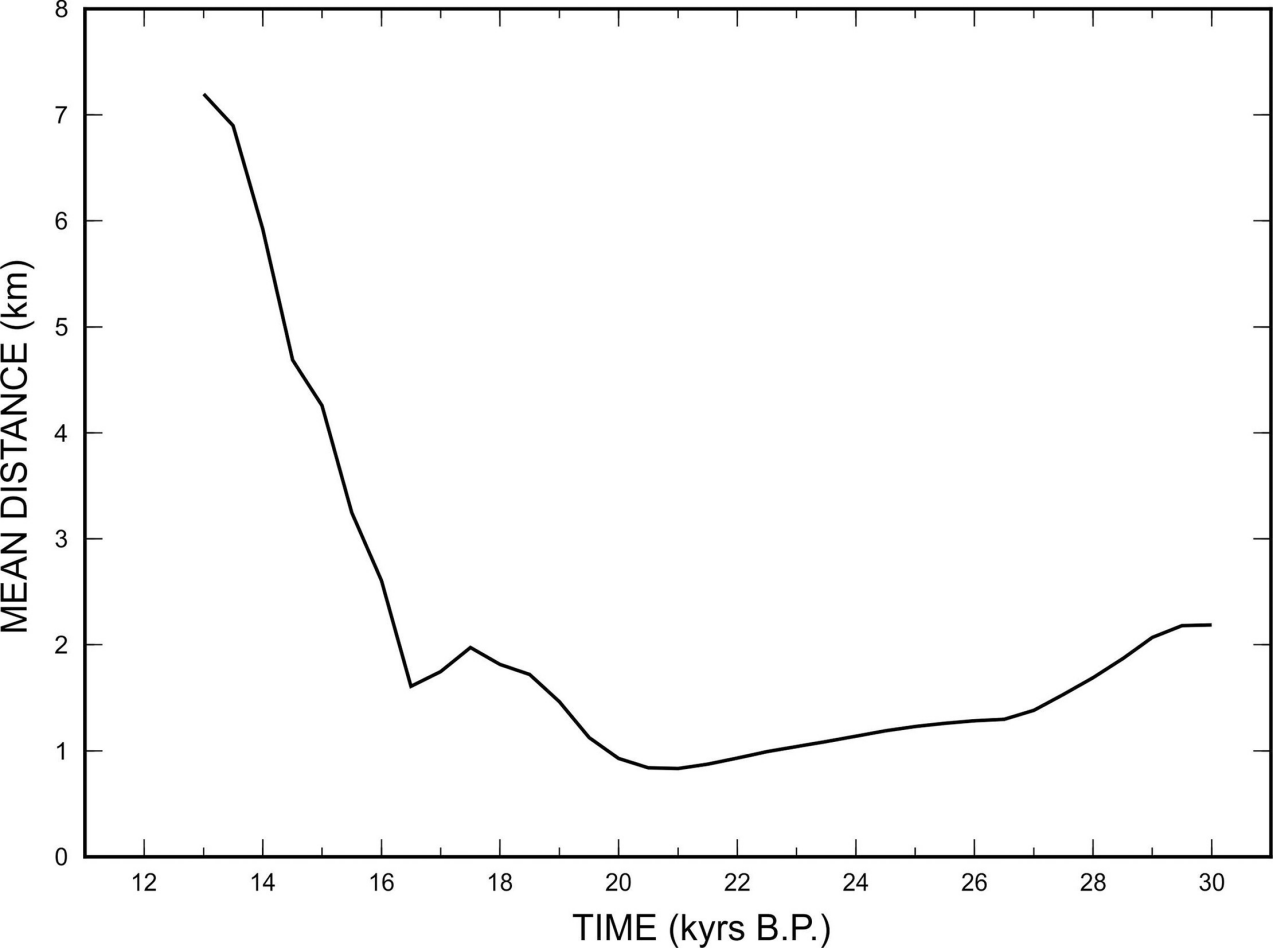
Distances between points derived from the GIA model and points derived from the interpretation of seismic data. It is noted that at 21 kyrs B.P., the average differences between the points are minimal, which means that the beginning of the marine transgression took place from 21 kyrs B.P.

However, it has been verified that most of the points mapped through the interpretation of seismic data fall along the palaeo-coastline drawn for the 21 kyrs B.P. (Fig 10). In this time frame, in fact, the average distance between the points (in latitude, longitude) derived from the seismic interpretation and those derived from the model, is minimal. This result makes it possible to determine, albeit indirectly, the beginning of the gradual rise in sea level following the LGM, in the absence of calibrations deriving from direct sampling of sediments immediately present above the erosional unconformity.

## Implications

The reconstruction of the ancient coastlines derived from the GIA model adopted in this study allows us to visualize how the geography has changed over time as a consequence of the relative sea-level rise, and provides clues on how the landscape of the coastal environment has been transformed. In scarcely mapped marine regions, these models represent the only way to describe the coastline evolution through time. One of the most suggestive applications of these palaeogeographic reconstructions is linked to underwater archaeology.

The global event of the sea-level rise has determined a significant modification of the previous geography, with the progressive retreat of the coastlines [57, 58]. Lowland areas and shallow shelves were particularly affected by this event, because rapidly invaded by sea water. In addition to environmental and morphological changes, this event has undoubtedly also profoundly changed the distribution of ancient human settlements and triggered large-scale population dispersals and migratory processes [59]. This is particularly the case of the Mediterranean basin because since ancient times it was a privileged way of communication between the human communities living on its coasts. However, unequivocally associating the abandonment of pre-existing settlements with the progressive sea-level rise, is an extremely complicated and often difficult task. This is due to the lack of sufficient archaeological information of the now submerged areas, and to the scarcity of geophysical data that unequivocally constrain the process of drowning the platforms. The models that reconstruct the trend of coastal lines over time are extremely useful as they provide a time-lapse of the evolution that has undergone a specific area. The degree of reliability of these models depends on the number and quality of geophysical information available, such as detailed bathymetric maps and high-resolution seismic profiles, from which it is possible to recognize the morphological elements associated with marine transgression.

Sea-level rise changed the physical landscape, inundated coastal sites, and created isolated environments in the form of new islands, bays and straits, which would have been the site of human occupation in the antiquity. Most of what we know today about the ancient civilizations is largely attributable to archaeological studies carried on land, while still few and sparse are the research conducted in now submerged areas. Despite the technologies for underwater exploration, mapping, and modeling have greatly improved in recent decades [60], the systematic investigation of continental platforms for archaeological purposes is still extremely scattered. In recent years, however, some initiatives and coordinated projects have been launched, such as the project *SPLASHCOS*, which collected data on over 2,500 archaeological submerged sites on the European continental platforms [61].

According to Bailey et al. [62], coastal habitats are amongst the most attractive for human settlement, and coastlines and seaways not as barriers but as gateways to human movement and contact, from early hominid dispersals to the rise of the great coastal and riverine civilizations. In addition, a growing body of evidence shows that ancient people regularly and readily occupied and/or accessed many smaller islands for both terrestrial and marine resources, privileging within an archipelago, an earlier occupation on smaller islands versus larger ones [63]. Very often, these habitats have been considered to be among the most marginal of environments.

The melting of the polar ice caps has released large quantities of fresh water into the oceans, starting from the end of the LGM. As a consequence of this phenomenon, sea level has increased at an average rate of 10 mm/yr during the period 19–7 kyrs B.P. Laboratory analysis and dating in some coral reefs of tropical oceanic islands revealed the presence of specific episodes of sudden and rapid increase in sea level—known as “melt-water pulses” (MWP) [64–66]. These episodes of rapid sea-level rise, which reached rates of up to 60 mm/yr for periods of time of 300–500 years, have led to a very rapid flooding of continental platforms. The curves of sea-level variation calculated for the Italian seas showed only slight variations in the sea-level rise after the LGM, unlike what is seen for the oceanic areas [4], and this suggested the absence of dramatic episodes of sea level rise such as MWPs. In contrast, recent bathymetric and seismic-stratigraphic studies in some sectors of the central Mediterranean have highlighted the presence of some specific submerged morphologies, such as palaeo-coastal cliffs, depositional terraces and barriers [67–70] most likely developed as a result of episodic and rapid rises in sea level, comparable to those of the MWPs. For example, in the shallow banks of the Adventure Plateau, the analysis of the Chirp profiles [18] showed that the rate of sea-level rise during the period between 11,500 yrs B.P. and 11,200 yrs B.P. was ~60 mm/yr, a value very close to the one estimated for the MWP-1A in the Sunda shelf [71]. This means that over a period of only ~300 years, the rise in sea level has significantly changed the shape of the former islands of the Adventure Plateau, progressively reducing their size, and making them gradually disappear.

The recognition of episodes of dramatic sea-level rise which led to rapid changes in coastal configuration and shoreline position has significant implications. That rate of change would be noticeable by humans living in coastal areas during a single generation, and in some cases well discernible at the annual scale, particularly in low-lying areas and especially where coastal resources were a considerable source of food, fuel and other aspects of economy. These rapid changes have heavily impacted on the distribution of human settlements, eventually forcing site abandonment and migrations.

## Conclusions

We have analyzed a large data-set of high-resolution seismic profiles (Chirp profiles) and bathymetric maps (Multibeam data) acquired in the NW sector of the Sicilian Channel to identify the stratigraphic markers of the marine transgression which followed the LGM, and to map the “wave-ravinement surface”, i.e., the surface resulting from wave erosion during the post-LGM sea-level rise. It provides the geometric constraints (i.e., the geographic location) for the palaeo-shorelines. The locations of these post-LGM seismic horizons individuated from the Chirp data have been then compared with the palaeo-coastlines produced by a GIA model for different time frames, in order to properly calibrate, through best-fitting, the initiation of the marine flooding immediately following the LGM. The coastline drawn for the 21 kyrs B.P. time frame represents the best fitting with the locations identified from high-resolution data, except for the marine sector between Mazara del Vallo and Sciacca, where data analysis has revealed a Quaternary tectonic vertical uplift of ~0.6 mm/yr. This part of the Sicilian Channel is traversed by a lithospheric-scale tectonic lineament characterized by transpressive movements and formation of folded structures. In addition, the shape of the bathymetric profiles acquired in this sector manifest significant difference with respect to the profiles acquired along the eastern margin of the tectonically stable Adventure Plateau, where the steep slope associated to the post-LGM transgression is well preserved.

The application of a GIA models in the study area, where geophysical data are available, has permitted to reconstruct with a good approximation the evolution of the progressive retreat of the shoreline and to identify those areas characterized by vertical tectonism. At the same time, the GIA modernization that we have performed has allowed to visualize the changing landscapes across a significant part of the Sicilian Channel, even where geophysical data are not available. One of the most attractive implications of our modeling of the ancient coastline evolution is linked with the reconstructions of the distribution of ancient human settlements, considering that the sea-level rise followed the LGM has severely modified the previous landscape, and has determined the conditions for human dispersals and large-scale migrations.
